# Epitaxial Cu_2_O Thin Films Deposited from Solution: the Enabling Role of Cu Diffusion into the GaAs Substrate

**DOI:** 10.1021/acsami.4c16485

**Published:** 2025-01-15

**Authors:** Shir Gefen, Taissia Rudnikov-Keinan, Alexander Rashkovskiy, Vladimir Ezersky, Nitzan Maman, Noy Zakay, Mariela J. Pavan, Yuval Golan

**Affiliations:** a Department of Materials Engineering, Ben-Gurion University of the Negev, Beer-Sheva 8410501, Israel; b Ilse Katz Institute for Nanoscale Science and Technology, Ben-Gurion University of the Negev, Beer-Sheva 8410501, Israel

**Keywords:** cuprous oxide, thin films, solution deposition, epitaxy, copper diffusion, substrate corrosion etch, redox reactions

## Abstract

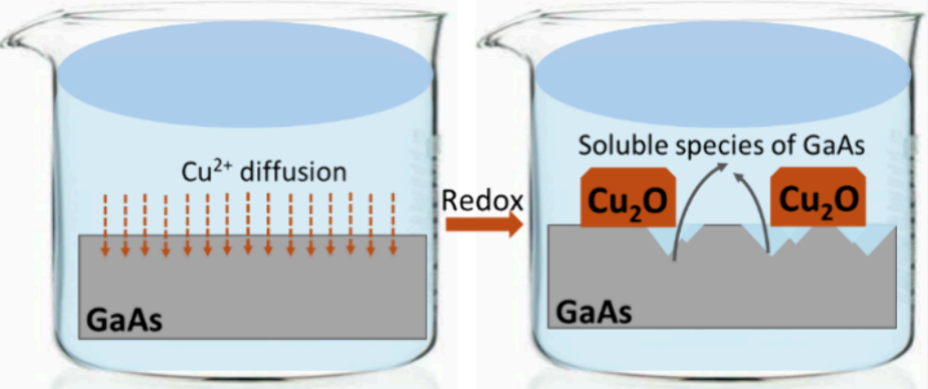

Cuprous oxide (Cu_2_O) thin films were chemically deposited from a solution onto GaAs(100) and (111) substrates using a simple three-component solution at near-ambient temperatures (10–60 °C). Interestingly, a similar deposition onto various other substrates including Si(100), Si(111), glass, fluorine-doped tin oxide, InP, and quartz resulted in no film formation. Films deposited on both GaAs(100) and (111) were found alongside substantial etching of the substrates. The etching of GaAs(100) was uneven, resulting in pyramid-like vacancies, while for GaAs(111), the etching was more even and resulted in a flat interface. X-ray diffraction measurements indicated highly preferential (110) growth of Cu_2_O regardless of GaAs substrate orientation, while TEM and a selected area of electron diffraction pointed out epitaxial growth on both substrates. X-ray photoelectron spectroscopy confirmed the diffusion of copper ions into the GaAs up to depths of 20 nm and the formation of intermediate phases at the interface. Raman spectroscopy indicated high structural quality of the films and showed good agreement with TEM and XRD results and Raman shifts corresponding to Cu_2_O, with no frequencies typical of CuO. The GaAs substrate appears to play a critical and unusual role in the deposition of Cu_2_O thin films on GaAs, which allows for growth of Cu_2_O in a previously unreported mechanism.

## Introduction

In recent years, cuprous oxide (Cu_2_O) has been gaining attention as a candidate material for applications in photovoltaics^1^ and catalysis^2^ and photocatalysis^2,3^ due to it being a nontoxic, cheap, and naturally abundant p-type semiconductor with a direct bandgap of 2.17 eV.^4,5^ As an oxide, it is highly stable in various environments, including highly alkaline solutions. To harness the potential of this material, a method is needed to easily and inexpensively form single-phase crystalline thin films with controlled orientations. Physical deposition methods such as evaporation,^6^ thermal oxidation,^7^ and magnetron sputtering^8^ were all used to create thin films of Cu_2_O; however, these methods are less viable for large-scale operations due to their relatively higher costs, expensive machinery, and high temperatures required. Chemical deposition methods such as successive ionic layer adsorption and reaction (SILAR),^9−11^ spray pyrolysis,^12^ sol–gel processing,^13^ electrodeposition,^14,15^ and chemical solution deposition (CD)^16^ are more cost-effective yet most methods resulted in nonuniform, low-quality, and less directional films.

Phase-pure Cu_2_O is a highly desirable material for its optoelectronic properties, including a direct bandgap of 1.90–2.50 eV, high mobility (μ ∼100 cm^2^/V s), and large minority carrier diffusion length (Lp ∼1–2 μm). It is also useful as an absorber material to realize efficient thin photovoltaic cells based on inexpensive, nontoxic, and earth-abundant metal oxides.^17^ Cu(I) is inherently less stable than both Cu(II) and Cu(0) in both atmospheric and aqueous environments.^17−19^ This instability poses significant challenges in the synthesis of phase-pure Cu_2_O, as the formation of CuO or Cu impurities is often difficult to avoid. These impurities have been demonstrated to adversely affect the performance of junction-based optoelectronic devices based on Cu_2_O.^11,17^ Consequently, the development of Cu_2_O thin films with high phase purity and desirable properties has been a long-standing area of research activity.

Among these techniques, the CD of thin films is cost-effective, straightforward, and suited for industrial scale-up.^20−23^ A wide range of semiconductor compounds was successfully deposited using CD including PbS,^24−26^ PbSe,^24^ CdS,^21^ CdSe,^27^ π-SnS,^28^ and γ-SnSe.^29^ In CD, a substrate is introduced into a reactor containing an appropriate solution in order to precipitate thin films in a controlled chemical reaction. Substrates typically used in the CD method include GaAs,^21^ Si,^30^ quartz, InP,^31^ glass, and others.^21^ By controlling deposition parameters such as deposition duration, temperature, pH, and solution composition, film morphology can be controlled, varying from individual nanocrystals to epitaxial monocrystalline films.^32,33^ CD of Cu_2_O and CuO was previously reported on fluorine-doped tin oxide (FTO) and glass substrates using elaborate processes requiring vacuum systems and high temperatures and resulted in films with multiple orientations.^16,34−38^ Cu_2_O/GaAs heterostructures are important in solar cell technology. Based on previous reports, computational studies have pointed out to impressive efficiencies predicted for Cu_2_O/GaAs dual junction solar cells, reaching 30.08%.^39,40^ Moreover, Cu_2_O films on FTO can be applied as catalysts for CO_2_ photoelectric reduction.^41^ Cu_2_O microcrystals onto silicon are highly promising for enabling new integrated photonic device architectures relevant for quantum information processing and quantum sensing.^42^

In this work, we demonstrate for the first time the epitaxial growth of single-phase thin films of Cu_2_O on GaAs using a simple three-component solution at ambient conditions and with no buffer layer and no external electric field. Previous studies depositing Cu_2_O using CD resulted in poor coverage of the film,^43^ a mixture of copper and copper oxides,^44^ and various film orientations.^43,45^ This work shows for the first time epitaxial films of Cu_2_O, containing no other phases of copper oxide deposited using CD. We highlight the unique, complex, and completely new and previously unreported deposition mechanism that involves rapid diffusion of copper cations into the GaAs substrate and discuss the galvanic processes and other key issues arising from the reactivity of the substrate.

## Experimental Details

### Materials

Cu(NO_3_)_2_ (Sigma, 99.999%), KOH (Sigma, ≥ 98%), sodium citrate dihydrate (Sigma, ≥ 99%), acetone (Bio-Lab, 99.9%), and 2-propanol (Bio-Lab, 99.8%) were used without further purification. Distilled water (DIW) was obtained using a Millipore Direct Q3 (18.2 MΩf). GaAs(100) and (111) wafer substrates (±0.1° miscut) undoped, epi-polished on both sides, were purchased from Geo Semiconductor (UK) and manufactured by AXT, Ltd. For control experiments, Si(100) and (111) wafer substrates (±0.5° miscut), single-side epi-polished, (Semiconductor Wafer Inc.) were dipped in 25% hydrofluoric acid (Merck, ISO, Reag. Ph. Eur.) solution for 20 s to remove native oxide. Undoped InP(100) wafer substrates (±0.5° miscut) epi-polished on a single side, were manufactured by AXT, Ltd. Soda Glass substrates were Menzel Gläser microscope glass slides. Fused quartz substrates were purchased from Ted Pella Inc.

#### Solution Deposition of Cu_2_O Thin Films

Thin films of Cu_2_O were deposited by using chemical bath deposition (CD). Double side-polished, semi-isolated GaAs(100), GaAs(111)A, and GaAs(111)B wafers were used, as well as quartz substrates, glass substrates, Si(100) and Si(111) substrates, and InP(100) substrate. Before the deposition, the substrates were cleaved into 1 × 1 cm^2^ pieces and cleaned as follows. Si(100) and Si(111) substrates were treated with hydrofluoric acid (20%) for 20 s to remove the native oxide layer. Glass substrates were plasma cleaned using air plasma for 5 min to improve surface quality. GaAs, quartz, and Si substrates were cleaned for 10 min in acetone and 10 min in DIW and washed with isopropanol. Finally, the substrates were dried under a N_2_ jet. Deposition was carried out in a 100 mL beaker using a solution with final concentrations of 1.9 mM Cu(NO_3_)_2_, 16 mM KOH, and 2.7 mM sodium citrate dihydrate at temperatures of 10–60 °C with deposition times of up to 1 h.

#### Characterization Methods

##### X-ray Diffraction (XRD)

The crystallographic phase and orientation of the films were characterized using a Panalytical third-generation Empyrean powder diffractometer equipped with an iCore incident beam optics and a dCore detector. Data were collected in the θ/2θ scan runs for 7 min in a 2θ range of 25–65° for GaAs(100) and 29–65° for GaAs(111) using Cu Kα radiation (λ= 1.5405 Å) at 45 kV and 40 mA with steps of ∼0.033°. The ranges of 2θ = 31°–33° for GaAs(100) and 55–56° for GaAs(111) were not scanned due to the presence of intense 200 and 222 GaAs reflections from the single-crystal substrate, respectively.

##### High-Resolution Scanning Electron Microscopy (HR-SEM)

The morphology of the films was investigated by a FEI Verios XHR-460L FEG-SEM without coating the surface. Acceleration voltage was fixed at 10 kV. The film thickness was measured from cross sections, and the surface topography was observed in plane view.

##### Transmission Electron Microscopy (TEM) and High-Resolution TEM (HR-TEM)

Bright field (BF), dark field (DF) TEM, and selected area electron diffraction (SAED) were carried out using a JEOL JEM-2100F analytical TEM operating at an accelerating voltage of 200 kV. TEM lamella cross-sectional samples were prepared using a Thermo Fisher Helios G4 UC dual-beam apparatus. Energy-dispersive spectroscopy (EDS) was carried out using an Oxford Instruments X-Max 65T SDD detector. Line scan EDS analysis was done in STEM mode. A Thermo Fisher Helios G4 UC FIB/SEM tool was utilized for TEM cross-sectional sample preparation. A 30 kV gallium beam was employed to mill the area surrounding and beneath the region of interest. Utilizing the Easylift micromanipulator, a protected volume of approximately 12 × 2 × 7 μm^3^ was delineated and affixed to a molybdenum TEM grid. The sample was then polished and thinned. Final thinning of the TEM sample surface was accomplished using a low-kV beam (5 keV) to remove residual amorphous layers, resulting in a nominal sample thickness of about 80 nm. It is noteworthy that sample cutting was kept parallel to the crystallographic plane of the wafer flat to maintain sample orientation to the zone axis within the TEM.

##### X-ray Photoelectron Spectroscopy (XPS)

XPS measurements were conducted with a Thermo Fisher Escalab Xi instrument, equipped with a monochromated Al Kα (*h*ν = 1486.6 eV) X-ray source with a beam size of 500 μm and an Ar^+^ cluster ion gun. High-resolution Cu 2p, O 1s, Ga 3d, and As 3d spectra were recorded every 60 s with an analyzer pass energy of 20 eV and a scan resolution of 0.1 eV during the depth profiling. Samples were depth profiled using an Ar^+^ cluster gun with a primary beam energy of U_0_ = 8 kV and an arbitrary cluster size of 75 au. The cluster gun parameters were adjusted to prevent reduction of any metallic ions under ion bombardment. The estimated sputtering rate was 1.5 nm/min. All spectra were recorded under charge compensation conditions using both in-lens and external flood gun charge neutralizer. After collecting the spectra and fine alignment of the peak positions to the GaAs substrate reference values (19.1 eV for Ga 3d5/2 with cross-check of As 3d position at 40.8 eV), a curve fitting procedure was performed to quantitatively analyze and separate different chemical states of the elements by determination of corresponding peak positions.

##### Raman Spectroscopy

Micro-Raman spectra were measured with a confocal Horiba LabRam HR Evolution, equipped with a Syncerity CCD detector (deep-cooled to −60 °C, 1024 × 256 pixels). The excitation source was a Nd:YAG 532 nm laser with a power on the sample of 0.35 mW. The laser was focused on the sample with a ×100 objective (MPLN100X, NA = 0.9) to a spot size of about 0.7 μm. The measurements were taken using a 600 g mm^–1^ grating and 100 μm confocal hole. The Raman setup was calibrated before measurements using the 520.6 cm^–1^ silicon band as reference. LabSpec software (version 6.5.2.11) was used to operate the equipment and for data processing (baseline correction and deconvolution).

## Results and Discussion

**Figure 1:**
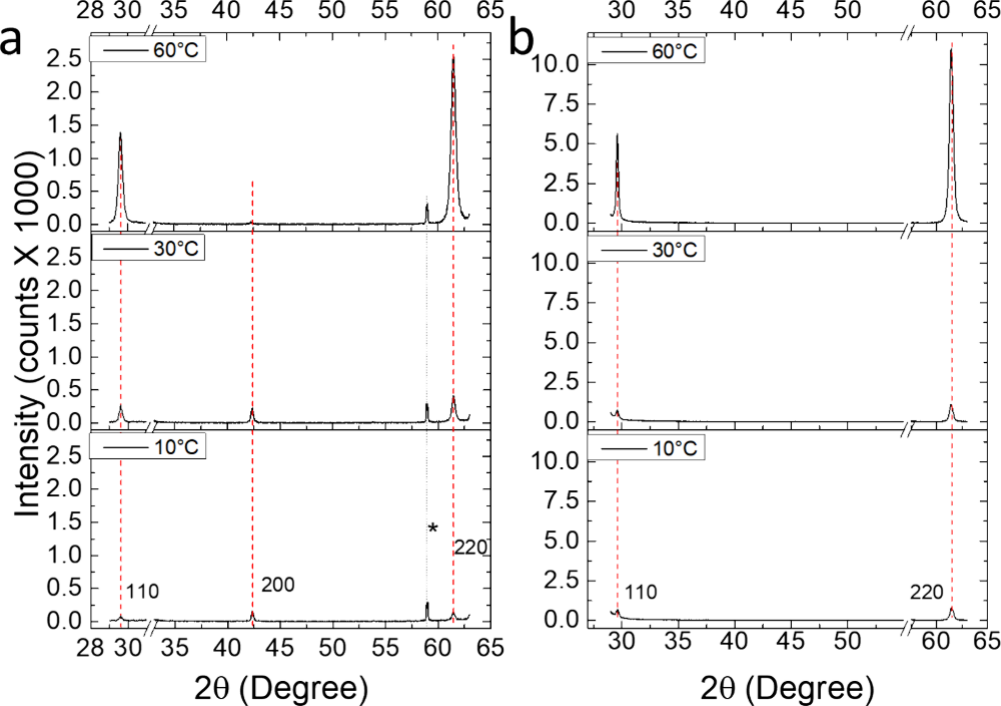
XRD analysis of Cu_2_O films grown from solution on GaAs at 10–60 °C for 1 h on (a) GaAs(100); (b) GaAs(111). Growth temperature is indicated in the upper left corner of each diffractogram. The peak at 2θ = 59° marked with an asterisk corresponds to the GaAs(400) Bragg reflection caused by residual Cu K_β_ radiation. Red dotted lines represent Cu_2_O JCPDS file no. 05–0667.

**Figure 2:**
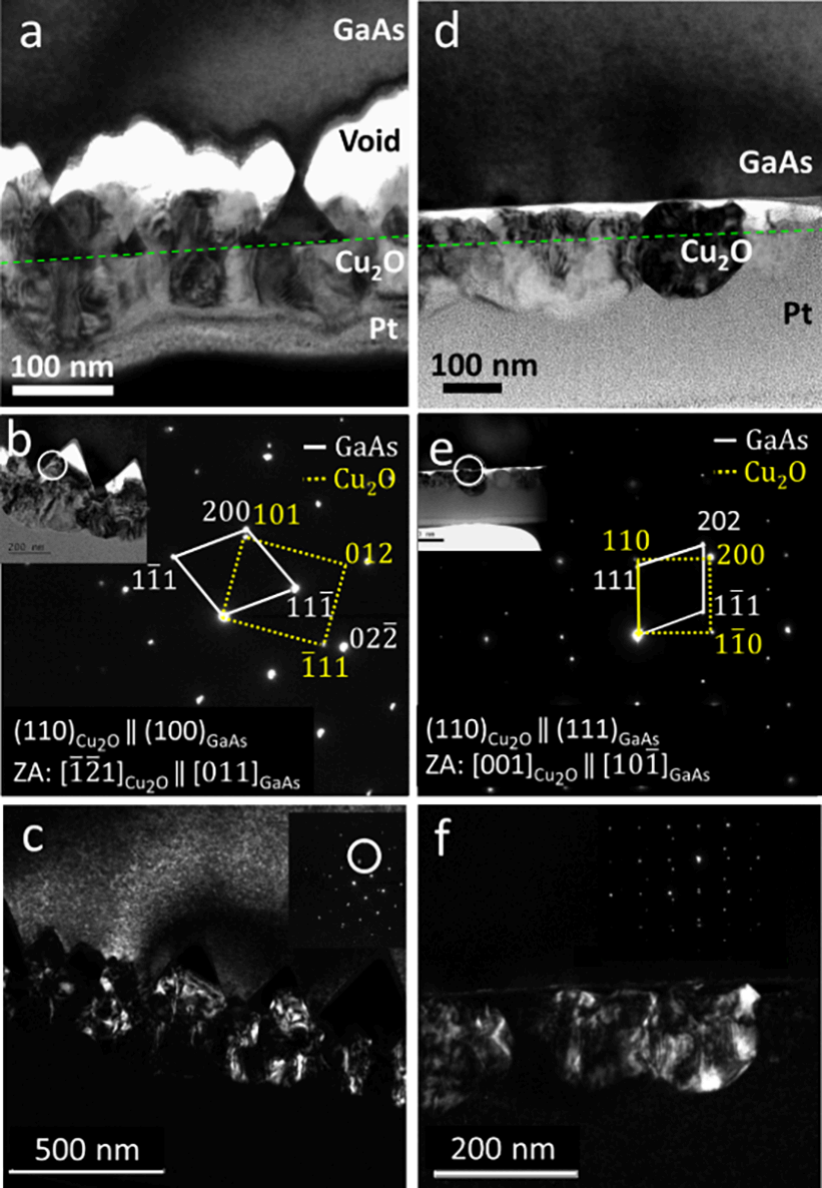
TEM of Cu_2_O thin films deposited from the solution onto GaAs substrates at 40 °C for 1 h. (a) BF TEM of a film deposited on GaAs(100). The green dotted line in panels (a) and (d) represents the original boundary between GaAs and Cu_2_O; (b) SAED pattern taken from the near-interface region (inset in the left upper corner) with (110)_Cu2O_∥(100)_GaAs_ and [1̅2̅1]_Cu2O_∥[011]_GaAs_ (zone axes) orientation relations; (c) DF-TEM of the solution-grown Cu_2_O film on GaAs(100); (d) BF TEM of a film deposited on GaAs(111)B; (e) SAED pattern taken from the near-interface region (inset in the left upper corner) with (110)_Cu2O_∥(111)_GaAs_ and [001]_Cu2O_∥[101̅]_GaAs_ (zone axes) orientation relations; (f) DF-TEM for the solution-grown Cu_2_O film on GaAs(111)B.

The XRD analysis shown in Figure 1 shows preferential growth of Cu_2_O (110) on both GaAs(100) and GaAs(111) substrates, in line with the results obtained from SEM. For growth on GaAs(111), the <110> out-of-plane growth direction is exclusively observed and is independent of growth temperature, with only peak intensity and width affected, indicating that temperature affects crystallinity, surface coverage, and layer thickness, yet not crystallographic orientation. For growth on GaAs(100), a weak 200 peak is observed at lower temperatures in addition to the main <110> out-of-plane growth direction. This growth direction is much less pronounced at higher temperatures, which exclusively show a <110> out-of-plane growth direction. TEM analysis in Figure 2 indicates epitaxial growth of the Cu_2_O film, along with prominent etching of the GaAs substrates. A detailed comparison of each diffraction peak at these temperatures for GaAs(111)B and (100) is provided in Figure S1.

On GaAs(100), the etching is quite uneven, resulting in triangle-shaped voids with angles ca. 55°, in agreement with the angle between <100> and <111> planes from the surface plane, and a resulting roughness of approximately 45 nm as can be seen in Figure 2a. A close lattice match between the *d*-spacings along the coaligned GaAs {100} and Cu_2_O {110} crystallographic planes can be seen through the SAED pattern shown in Figure 2b as well as in the inset in Figure 2c. Using dark field imaging (Cu_2_O reflection circled in Figure 2c), it is also observable that the etching is much larger as the film grows both into and out of the substrate, resulting in etching of approximately 170 nm as seen in Figure 2c. For GaAs(111), the etching is even, and it is possible to determine its occurrence mainly from the dividing line formed at the original interface, as indicated by the green dashed line, as can be seen in Figure 2d. This could be attributed to the difference in etch rate (ER) of different planes of GaAs.^35^ Co-alignment of the GaAs {111} and Cu_2_O {110} planes can be seen in Figure 2e as well as in the inset in Figure 2f. The dark field image taken from the <110> reflection of Cu_2_O shows grains at several different in-plane orientations (Figure 2f).

**Figure 3:**
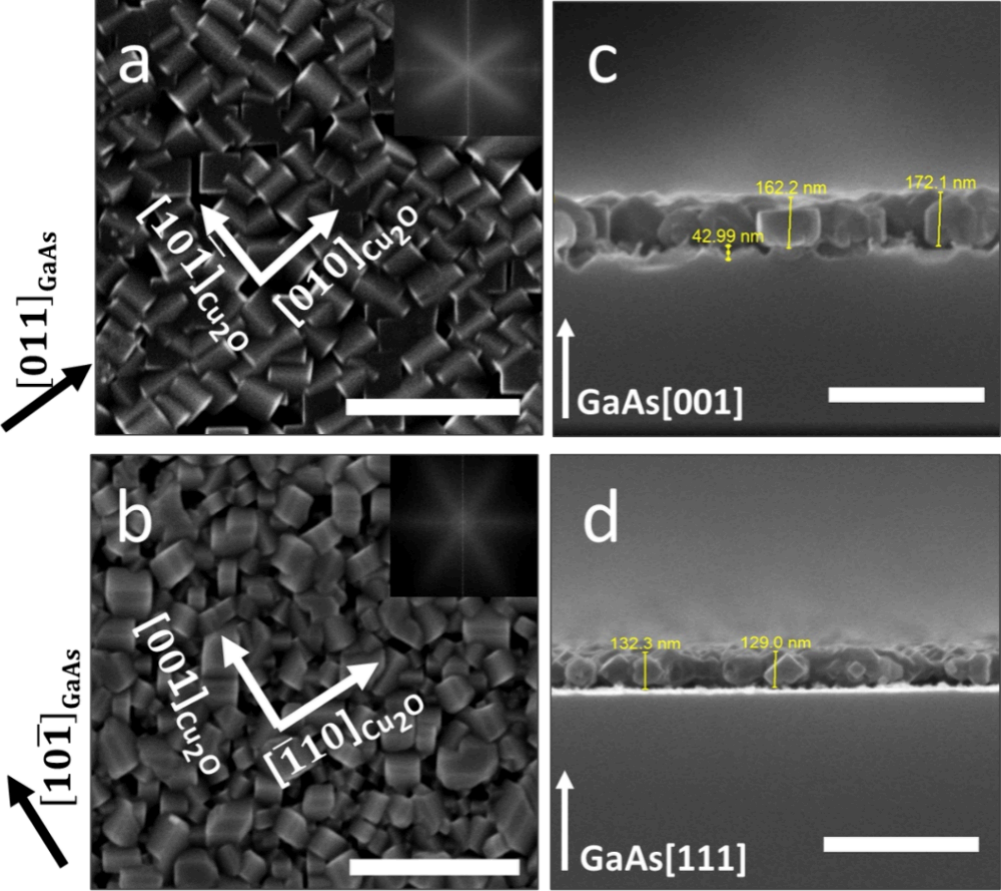
HR-SEM of Cu_2_O films grown from the solution onto GaAs substrates. (a) Plane-view image of Cu_2_O grown on GaAs(100); (b) plane-view image of Cu_2_O grown on GaAs(111); (c) cross-sectional image of Cu_2_O grown on GaAs(100); (d) cross-sectional image of Cu_2_O grown on GaAs(111). Insets in panels (a,c) show FFT of the SEM images, showing the highly directional film texture. Scale bar in all images corresponds to 500 nm.

The HR-SEM plane-view images in Figure 3a,b indicate the presence of continuous, polycrystalline Cu_2_O thin films with uniform substrate coverage. Additionally, the FFT analysis of SEM images clearly shows that the Cu_2_O grains are oriented along the <110> in-plane direction of the substrate (three observed orientations for Cu_2_O grains on GaAs(111)B are schematically illustrated in Figure S2), as shown in the insets of Figure 3a,b, respectively.^46^ Severe etching of the substrate surface is clearly observed following CD (Figure 3c). In the case of GaAs(100) substrate, etching is nonuniform with a typical damage depth of 40–45 nm, as detected by SEM on freshly cleaved wafers. Conversely, for GaAs(111), the existence of substrate etching cannot be detected using SEM and the interface is uniform (Figure 3d).

It is notable that the Cu_2_O thin film growth was exclusively observed on GaAs substrates. In control experiments carried out on other types of substrates, including Si, quartz (SiO_2_), FTO, InP(100), and soda glass, no film growth was observed.

**Figure 4:**
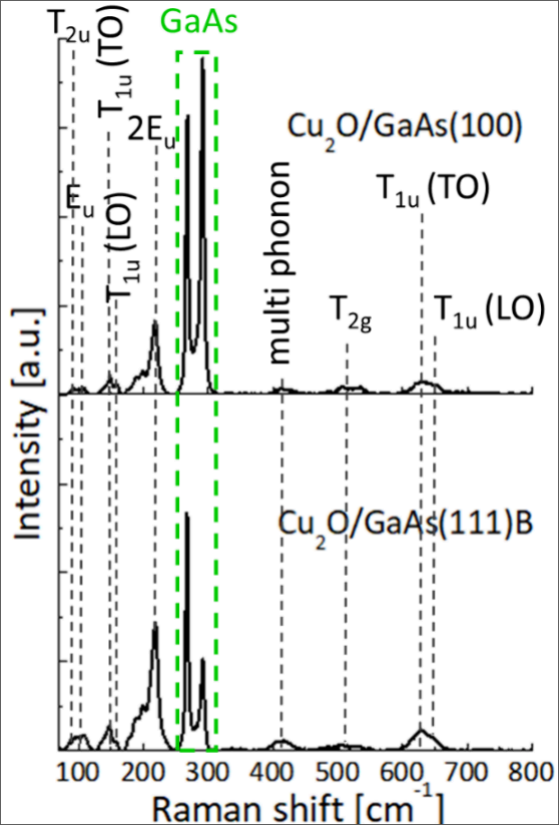
Typical Raman scattering spectra for Cu_2_O thin films grown onto GaAs(100) and GaAs(111) substrates at 60 °C for 1 h.

The Raman spectra for both samples (Figure 4) show the characteristic phonon frequencies of the crystalline Cu_2_O without specific peaks related to the CuO material (i.e., 299 and 346 cm^–1^),^47^ supporting the TEM results in Figure 3. The Raman spectra at 267 and 285 cm^–1^ correspond to scattering by transverse-optical (TO) and longitudinal-optical modes (LO) modes of the GaAs substrate.^48^ The peak assignment for Cu_2_O with their respective symmetry is shown in Table 1. The only Raman-allowed mode (*T*_2g_) is observed as a low intensity band at 505 cm^–1^. The Raman spectra are interestingly dominated by the presence of bands that are forbidden by symmetry rules, which were reported to be a consequence of point defects in the crystal, or by disruption of the selection rules under resonance conditions (*T*_2u_, *E*_u_, *T*_1u_, and 2*E*_u_ and combination bands).^49^ These defects, especially Cu split vacancies,^50^ which are likely responsible for intrinsic p-type conductivity of Cu_2_O films,^51^ produce a strong perturbation in the selection rules in the case of Cu_2_O, causing the vast activation of the Raman forbidden modes and the splitting of the *T*_2g_ band.^52−55^ Raman analysis confirmed pure crystalline Cu_2_O film formation during chemical growth from the solution, with typical p-type conduction behavior, earlier reported for Cu_2_O films grown by physical phase and electrodepositions techniques.^56,57^

**Table 1:** Symmetry Types and Characteristic Raman Shifts Observed in Raman Spectra of Cu_2_O Films Grown from the Solution onto GaAs(100) and GaAs(111) Substrates^44,47^

Mulliken symbol	Raman shift (cm^–1^)	nominal activity
*T*_2u_	91	silent
*E*_u_	103	silent
*T*_1u_ (TO)	147	infrared
*T*_1u_ (LO)	158	infrared
*T*_2u_ + *E*_u_	198	infrared
2*E*_u_	217	infrared
multiphonon mode	412	silent
*T*_2g_	505	Raman
*T*_1u_ (TO)	630	infrared
*T*_1u_ (LO)	650	infrared

**Figure 5:**
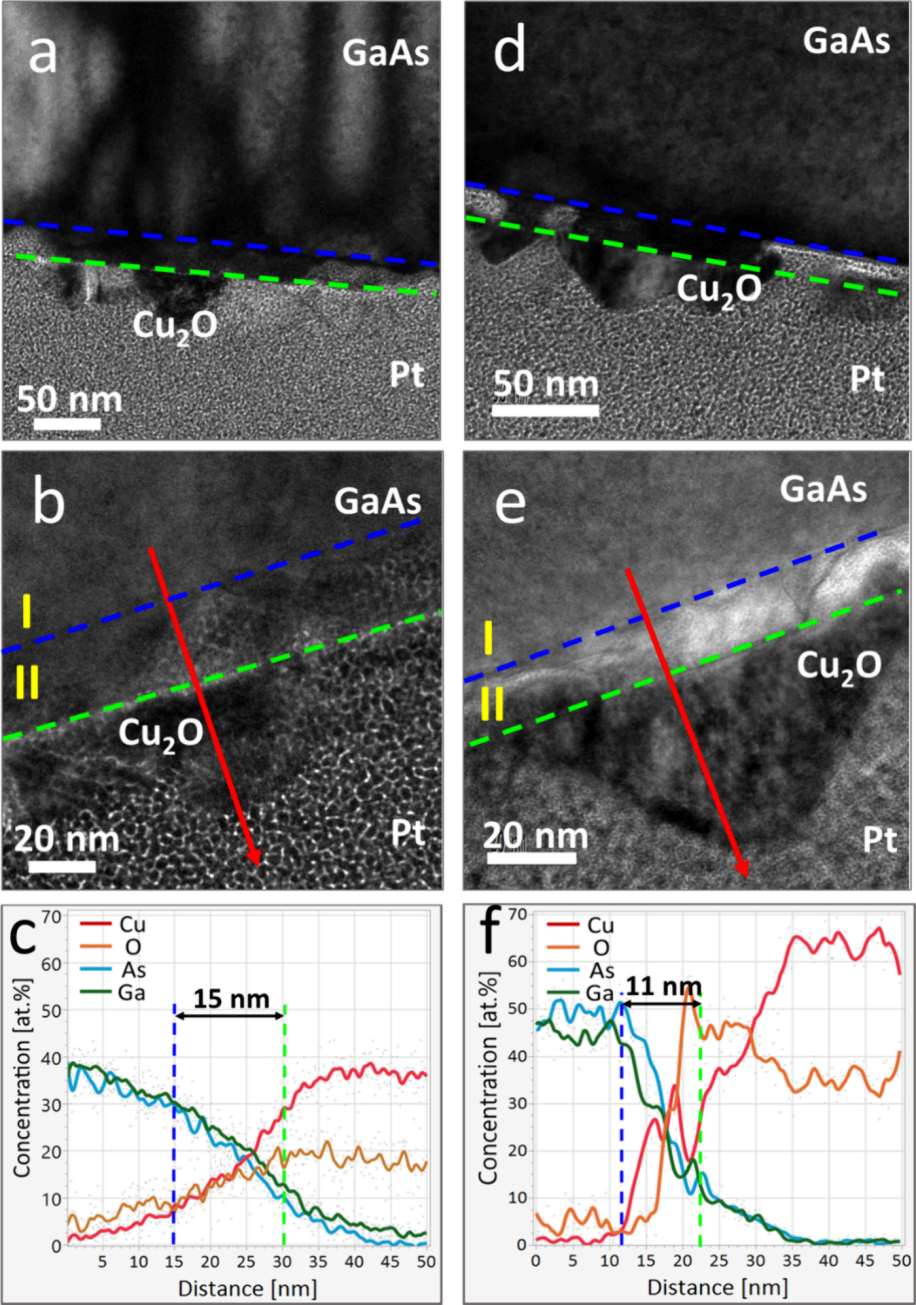
TEM analysis of the early stages of Cu_2_O grown from the solution onto GaAs at 50 °C for 5 min. (a,b,c) BF TEM and EDS line scan of Cu_2_O grown on GaAs(100) respectively; (d,e,f) BF TEM and EDS line scan of Cu_2_O grown on GaAs(111), respectively. The green dotted lines represent the original interface between GaAs and Cu_2_O while the blue dotted lines represent the resulting GaAs/Cu_2_O interfaces. The red arrows represent the EDS line scan path.

To better understand the mechanism of the epitaxial growth of Cu_2_O films on GaAs, the early stages of growth were investigated. The following deposition was carried out at 50 °C for a short time of 5 min. Etching of the substrate was observed by TEM for both GaAs(100) and (111), as can be seen in Figure 5a,d, respectively, and the identification of the Cu_2_O grains was established using TEM-SAED (not shown) and TEM-EDS. Line-scan EDS carried out along the red lines shown in Figure 5c,f clearly pointed out to the diffusion of Cu into the substrate, as can be seen in Figure 5b, e creating two regions: the original interface and the intermediate region, which includes both copper as well as GaAs. It is important to note that Mo TEM grids were used to prevent Cu from the grid from affecting the quantitative EDS analysis.

**Figure 6:**
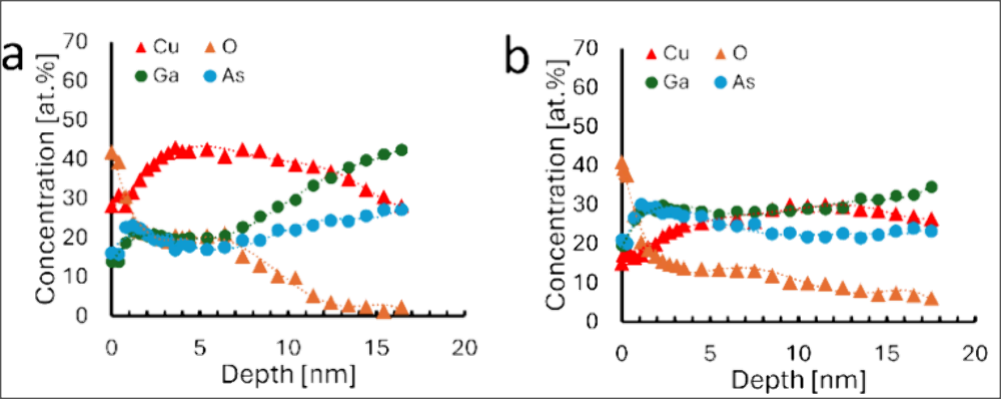
Atomic concentration of Cu, O, Ga, and As as a function of sputtering depth during XPS profiling for Cu_2_O thin films deposited from the solution for 2 min onto (a) GaAs(100); (b) GaAs(111).

The results shown in Figure 5 hinted at the importance of diffusion of Cu into the substrate for film growth. To further investigate the diffusion of Cu into the GaAs substrate at the early stages of film formation, XPS depth profiles were recorded for the same films after 2 min of substrate exposure into the solution. It can be seen from Figure 6a that the copper concentration within the substrate is constant in the region of 3–7 nm for GaAs(100) while it is linearly decreasing for GaAs(111) up to a depth of ca. 10 nm (Figure 5b). Meanwhile, signals from spectral components related to GaAs show a dip for GaAs(100) in the region where the most intensive surface corrosion takes place.

It is seen that Cu^2+^ ions from the solution readily penetrate the GaAs substrate, forming chemical bonds with Ga and As, as seen from the position of the Cu 2p lines and formation of additional components in the Ga and As XPS spectra.

At the same time, there is no signal from oxygen, as evident from the absence of peaks in the O 1s region. Notably, the position of the Cu 2p line at the surface corresponds to Cu^+^ and the O 1s line shows a sharp structure with two lines, which correspond to the native oxide of GaAs and to the Cu_2_O film nucleation centers. Meanwhile, Ga and As also show features related to the formation of the GaAs oxide. The results shown in Figure 6a,b reveal that Cu ions are diffusing into the GaAs substrate during the CD process. Comparing the element distribution in GaAs(100) and GaAs(111) substrates, it is clearly seen that copper and oxygen are distributed differently. On one hand, the surface and subsurface layers of the GaAs(100) substrate are enriched with Cu at levels of more than 30 at. % up to a depth of 15 nm. On the other hand, oxygen other than the native oxide of GaAs was detected only in the Cu_2_O layer. For GaAs(111), up to 25 at % of Cu and larger concentrations of oxygen atoms (up to 8 at %) were found within the substrate even below 15 nm. These findings support the idea that Cu_2_O nucleation occurs by extraction of copper(I) to the damaged surface by redox process in the initial stages of growth, but due to the crystallographic orientation, this extraction is delayed for the (111)-oriented substrate compared to (100).

**Figure 7:**
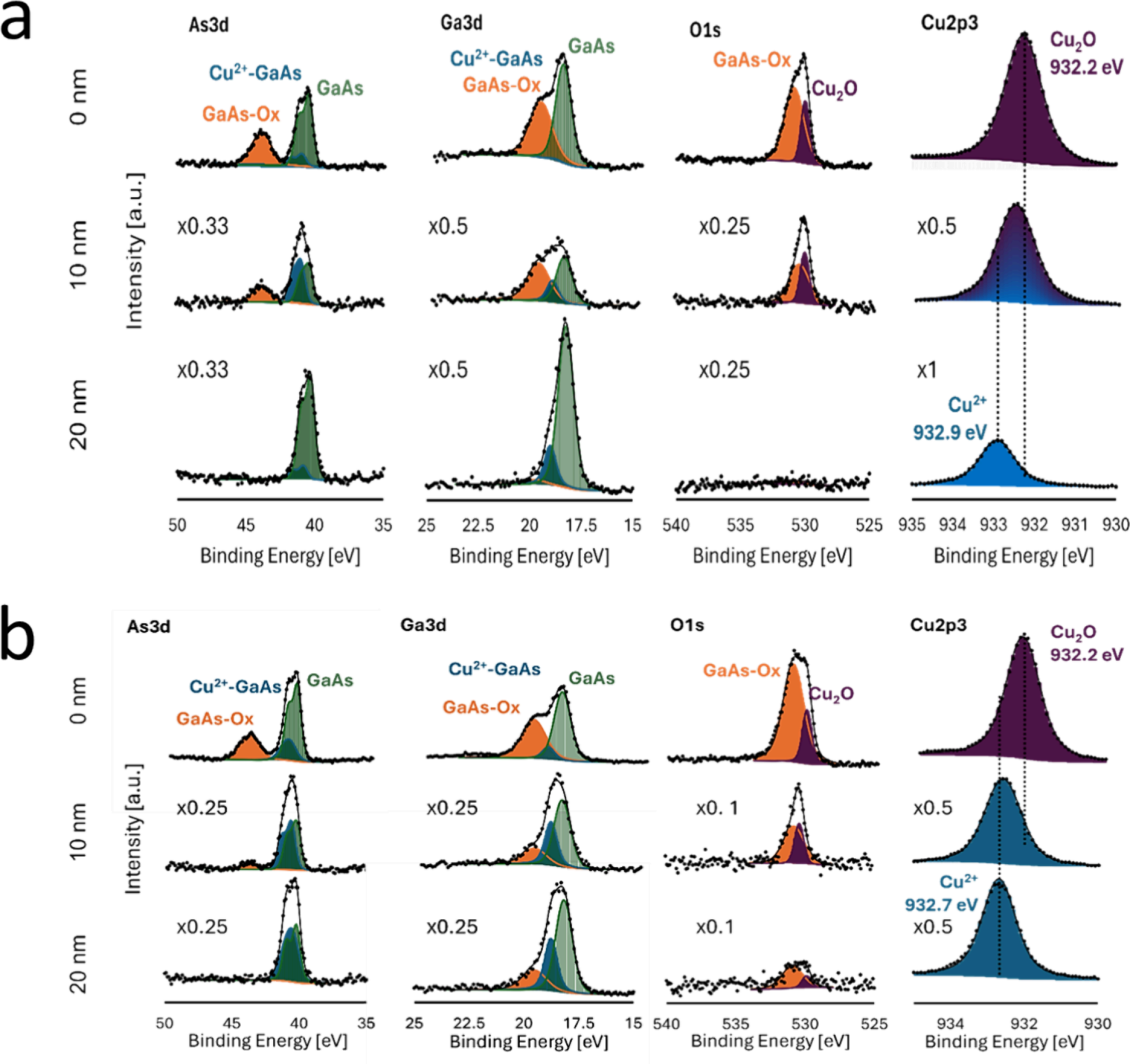
Deconvolution of XPS As 3d, Ga 3d, and Cu 2p core-level spectra onto individual states for Cu_2_O films deposited on (a) GaAs(100) and (b) GaAs(111), respectively. Spectra obtained from the surface (0 nm) and after profiling of the films to 10 and 20 nm depth are shown from top to bottom. Intensity multipliers are shown with respect to the corresponding peak height of 0 nm spectra.

**Figure 8:**
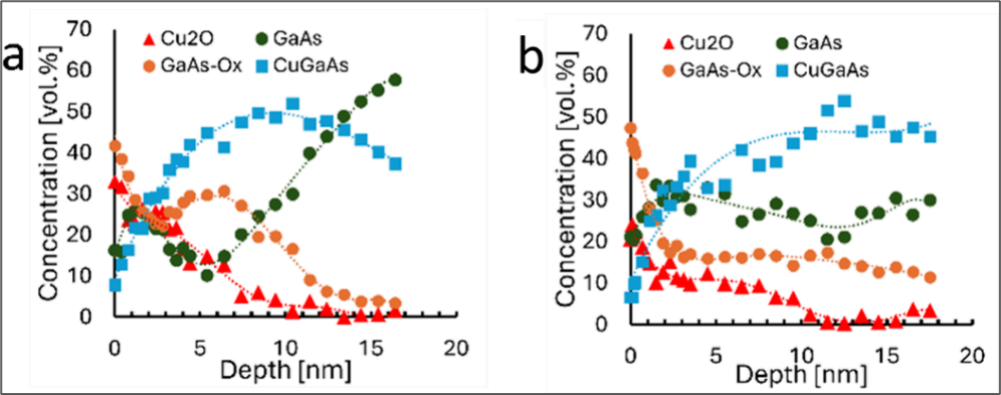
Corresponding distribution of the chemical states from deconvolution of high-resolution XPS spectra on (a) GaAs(100); (b) GaAs(111) substrates. (See Figure 6.)

Thus, during the first 2 min of deposition, only partial formation of Cu_2_O nuclei by the above mechanism occurs at synthesis temperature of 50 °C. Further calculations, based on the chemical shifts and curve fitting analysis (Figure 7a,b), allowed attributing chemical states to the different phases: Cu_2_O, GaAs, interfacial phase of Cu–Ga–As, and native GaAs oxide and estimating their concentration profiles over the thickness of the samples. From the Cu 2p XPS spectra of both samples, it is seen that on the surface, the Cu^+^ state is prevalent, which corresponds to the formation of the Cu_2_O film. At a depth of 10 nm from the surface in the film, grown on a GaAs(100) substrate, the intermediate state of Cu was detected, corresponding to the mixed signal of the Cu atoms belonging to the film (Cu^+^) and diffused into the substrate (Cu^2+^). Meanwhile the sharp leap between the Cu states from Cu^+^ (932.2 eV) to Cu^2+^ (932.7 eV) is found for the films grown on the GaAs(111) substrate, which confirms the results of STEM/EDS and XPS depth profiling. The corresponding depth profiles are shown in Figure 8a,b for GaAs(100) and GaAs(111), respectively. Extraction of the Cu ions from GaAs is slowed for the GaAs(111) substrate; thus, more uniform corrosion of the surface occurs and the Cu_2_O growth rate increases. By analyzing the Ga 3d, As 3d, and Cu 2p core-level spectra, it can be noted that Cu^2+^ ions [binding energy (BE) of 932.9–932.7 eV] within the GaAs substrate surface are forming chemical bonds with Ga and As, as can be seen from the position of Cu 2p lines and formation of additional components on the Ga 3d and As 3d XPS spectra labeled “Cu-GaAs”. Meanwhile, there is no signal from oxygen, evidenced by the substantial decrease of the O 1s intensity of the O 1s at depths of about 20 nm. On the other hand, the position of Cu 2p line at the surface corresponds to Cu^+^ (BE = 932.2 eV) and the O 1s line has a sharp structure with two lines, which correspond to GaAs native oxide and Cu_2_O film nucleation centers. In addition, Ga and As also show features related to GaAs oxide formation due to incomplete coverage of the surface by Cu_2_O nuclei in the early stages of the film formation. To understand the root cause of the effect of the solution on the GaAs surface during the deposition of Cu_2_O, each parameter of the system was tested separately. The substrate material itself, as well as all combinations of the solution components, was examined with further XPS analysis. The Auger analysis is provided in Figure S3.

Comparative XPS analysis of the Cu ion behavior during the early stages of Cu_2_O synthesis is shown in Figure 7 by measuring samples of GaAs(100) and GaAs(111) after 2 min of treatment in the deposition solution (Cu(NO_3_)_2_ + citrate + KOH). As a control experiment, Si(100) and InP(100) substrates were used, as well as GaAs exposed to the Cu(NO_3_)_2_ (single component) solution, and Cu(NO_3_)_2_ + citrate and Cu(NO_3_)_2_ + KOH binary solutions were also used to separately examine the role of each of the reaction components on the surface chemistry.

**Figure 9:**
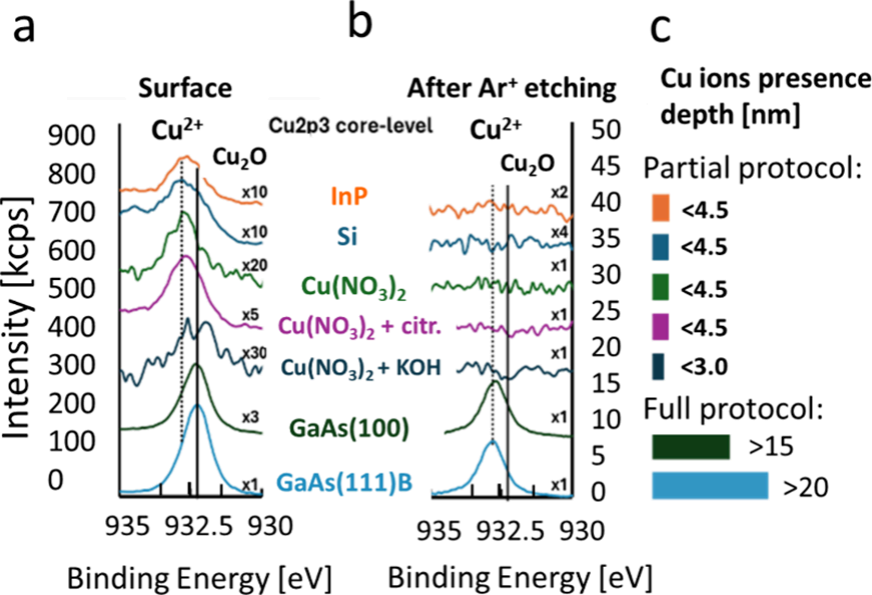
HR-XPS spectra for Cu 2p3 from the (a) surface and (b) at a depth, as specified in panel (c). From top to bottom: Full Cu_2_O deposition protocol was applied for InP(100) and Si(111) substrates; partial deposition protocol was applied for GaAs(100) in a solution of only Cu(NO_3_)_2_ (single component); binary solutions of Cu(NO_3_)_2_ + citrate and Cu(NO_3_)_2_ + KOH; and Cu_2_O deposited on GaAs(100) and (111) from the ternary solution (the deposition bath solution including all three components). All data shown was taken from samples after 2 min of deposition (interrupted growth).

It is seen that while Cu signal was detected from the surface for every substrate (GaAs, Si, and InP) (Figure 9a), after Ar^+^ ion etching, the only full Cu_2_O synthesis protocol on GaAs(100) and (111) resulted in the preservation of the Cu signal at reasonable depths of approximately 15–20 nm, as shown in Figure 9b. The thickness of the detectable Cu-containing layer was almost an order of magnitude smaller than that after a full protocol deposition with GaAs substrates. Moreover, it is seen that only in the case of the proposed scheme of the synthesis, which causes Cu penetration into GaAs, Cu^+^ oxidation states are formed, while in the case of other substrates and a limited number of precursors, only Cu^2+^ species were detected. The reaction previously suggested to govern the deposition was the redox reaction between the Cu^2+^ ions and the hydroxide (OH^–^) ions:^16^
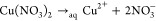
1
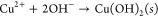
2
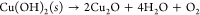
3

However, this process should be independent of the substrate used and would result in growth on all substrates examined in this experiment, albeit at different deposition rates and varying resulting morphologies. Therefore, it is obvious that the above reactions are insufficient to explain the growth obtained in this work.

It was originally expected for Cu_2_O deposition to be successful on InP and Si substrates, as the small lattice mismatch (*a*_Cu2O_ = 4.260 Å, *a*_InP_ = 5.869 Å, *a*_Si_ = 5.430 Å) should allow for epitaxial growth of the material, with a mismatch <4% for both (110)_Cu2O_∥(111)_Si_ and (110)_Cu2O_∥(200)_InP_. However, epitaxial growth was not observed for substrates other than GaAs. From the above results, it can be summarized that the factor distinguishing GaAs from other substrates that most likely allows for Cu_2_O film deposition is the rapid and effective diffusion of copper into the GaAs substrate in the early stages of the deposition.

**Figure 10:**
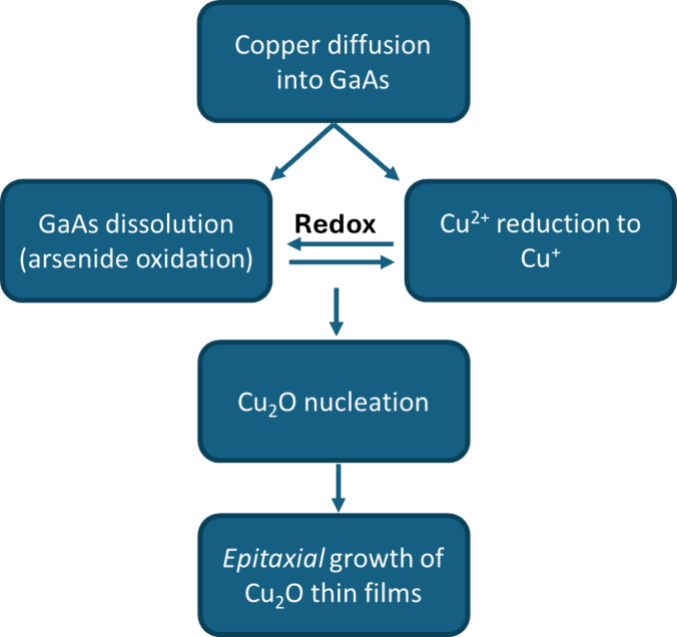
Schematic process of the Cu_2_O thin film deposition on GaAs.

A few different deposition mechanisms can be considered in the formation of the Cu_2_O film on the GaAs. The governing chemical reaction in this experiment seems to be a redox reaction between the GaAs substrate and the Cu^2+^ ions in the solution. The suggested sequential process is depicted in Figure 10.

As seen in Figure 9, the diffusion of copper ions into GaAs is substantial, while diffusion into all other substrates using the same experimental parameters was not observed. This, coupled with the fact that the etching of the substrate occurs only in GaAs and only in the presence of Cu^2+^ cations in an alkaline (OH^–^) environment, indicates that all three components are required for the reaction. Moreover, little to no diffusion of Cu^2+^ is observed in the absence of citrate in the substrate. This is likely due to the rapid reaction between the copper ions and hydroxide ions, resulting in Cu(OH)_2 (s)_ formation. The rapid hydroxide formation inhibits the diffusion of Cu^2+^ onto the substrate. The presence of sodium citrate acts as a complexing agent for the Cu^2+^, slowing down hydroxide precipitation^58,59^ and allowing for copper to diffuse into the substrate. Literature indeed shows that Cu readily diffuses into GaAs.^50,51^ However, these results occurred at annealing temperatures above 550 °C. At lower temperatures, it was shown by Kowalczyk et al. that Cu does not diffuse through either Ga_2_O_3_ nor As_2_O_3_.^60^

However, other reports discuss the instability of GaAs oxides under alkaline environments.^53^ This instability of oxides at low temperatures appears to allow for copper ion diffusion into GaAs even at ambient temperatures, as seen in our results. While the diffusion of copper ions into GaAs from water-based solutions has been discussed in the past,^61^ its effect as a precursor for Cu_2_O thin film formation via etching of the substrate was previously unknown. Comparing the diffusion coefficients between different substrates, the diffusion coefficient in GaAs at our deposition temperatures is more than 3 orders of magnitude higher than in the other substrates. The rapid diffusion has been attributed to the interstitial movement of Cu atoms.^62−66^ The diffusion of Cu^2+^ into GaAs creates a buffer layer between the film and the substrate materials, which appears to improve chemical compatibility. The enabling redox couple includes the reduction of Cu^2+^ into Cu^+^, while GaAs undergoes oxidation into soluble Ga^x+^ and As^y+^ species, resulting in etching of the GaAs substrate by a galvanic etching mechanism. In the GaAs system, there are reports of galvanic etching of both *n*-GaAs and *p*-GaAs coupled to noble metals, even at room temperature. The corrosion process involves formation of Ga and As oxides, which are typically soluble and removed to the aqueous solution where the corrosion is taking place, giving rise to etched regions in the GaAs.^67,68^ The redox reactions can be described as follows:^69,70^
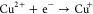
4
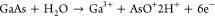
5

This galvanic cell formation allows for the subsequent growth of Cu_2_O until complete coverage of the GaAs substrate is achieved, preventing further Cu^2+^diffusion and thus terminating the reaction. At the same time, further growth of the film occurs from the solution and onto the existing film according to the reaction chain shown in eqs 1 and 2. As discussed previously, the difference in ERs of the different planes of GaAs resulted in uneven etching in GaAs(100) as it was readily etched along the <111> directions, as can be seen from the TEM results, which show an angle of ca. 55° between the etched and the original plane. For the same reason, the etching of GaAs(111) was harder to observe, as it was much more uniform along the preferred direction.

## Conclusions

In summary, CD was used to grow adherent and continuous thin films of Cu_2_O on various faces of the GaAs substrates. The orientation of the Cu_2_O thin films grown from the solution on GaAs(111) was independent of the deposition temperature at a temperature range of 10–60 °C. The orientation of the Cu_2_O thin films grown from solution on GaAs(100) was tunable by modifying the deposition temperature. At temperatures lower than 40 °C, Cu_2_O 200 peaks were observed, and at temperatures lower than 20 °C, 200 was the highest intensity peak, while at temperatures higher than 40 °C, only 110 diffraction peaks were observed, similar to films deposited on GaAs(111). Cross-section TEM analysis showed etching of the substrate, which was confirmed by XPS to occur due to diffusion of Cu^2+^ cations into the substrate and subsequent redox reactions, which result in the reduction of Cu^2+^ to Cu^+^ and simultaneous dissolution of the GaAs substrate. The remarkable diffusion of Cu^2+^ cations into the GaAs substrate and the associated redox reactions was shown to be crucial for the formation of the Cu_2_O film.

## Supporting Information Available

XRD results showing Cu_2_O deposited at different temperatures (10, 30, and 60 °C); a schematic illustration of the three possible orientations of Cu_2_O grains on the GaAs(111)B system; and the Cu LMM Auger line obtained from the surface of Cu_2_O films (PDF)
