# Supplementary material for: Epitaxial Cu2O Thin Films Deposited from Solution: the Enabling Role of Cu Diffusion into the GaAs Substrate
Source: ACS Appl Mater Interfaces. 2025 Jan 15;17(4):7066–75. doi: 10.1021/acsami.4c16485 (PMC11788980; doi:10.1021/acsami.4c16485)
Supplement: Supplementary file 1 — am4c16485_si_001.pdf [file am4c16485_si_001.pdf]

Supporting Information

Epitaxial Cu_2O thin films deposited from solution: The enabling role of Cu diffusion into the GaAs substrate

Shir Gefen, ‡ [a,b] *Taissia Rudnikov-Keinan,* ‡ [a,b] *Alexander Rashkovskiy,* [a,b] *Vladimir Ezersky,*[b] *Nitzan Maman,*[b] *Noy Zakay* [a,b] *, Mariela J. Pavan* [b] *and Yuval Golan* [* a,b]

[a] Department of Materials Engineering, Ben-Gurion University of the Negev, Beer-Sheva 8410501, Israel

[b] Ilse Katz Institute for Nanoscale Science and Technology, Ben-Gurion University of the Negev, Beer-Sheva 8410501, Israel

KEYWORDS: Cuprous oxide, thin films, solution deposition, epitaxy, copper diffusion, substrate corrosion etch, redox reactions.

* Corresponding author ; ygolan@bgu.ac.il

‡ These authors contributed equally to this work.

Additional XRD results showing Cu_2O 110, 200 and 220 diffraction peaks shown at 10,30 and 60 ℃ for GaAs(100),(111)b and Auger line of Cu_2O surface.

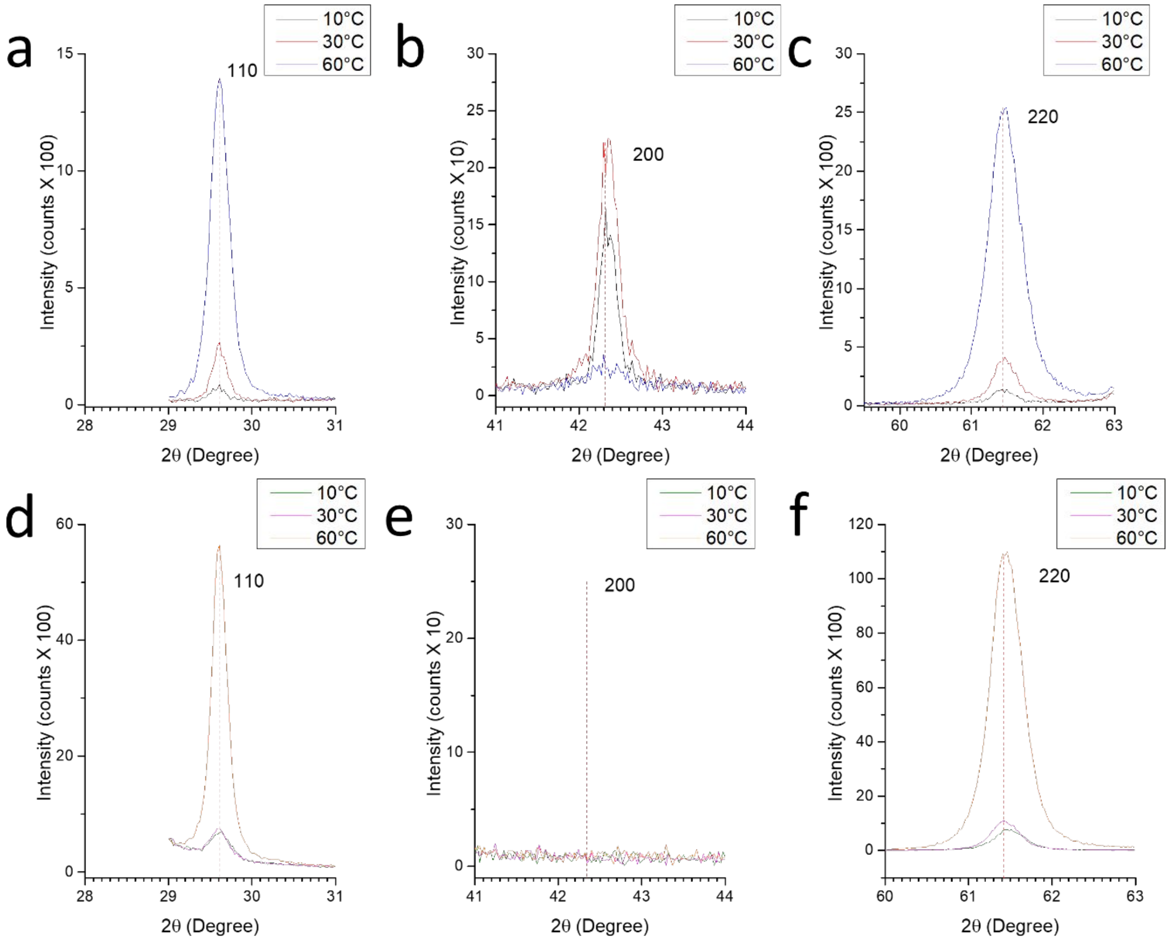

Figure S1. X-ray diffractogram patterns comparing Cu_2O films chemically grown from solution on GaAs at 10, 30 and 60 ^{0}C for 1 hr. (a-c) deposition on GaAs(100); (d-f) deposition on GaAs(111)B. For both substrates, the three Bragg peaks present in the diffractograms are 110, 200 and 220.

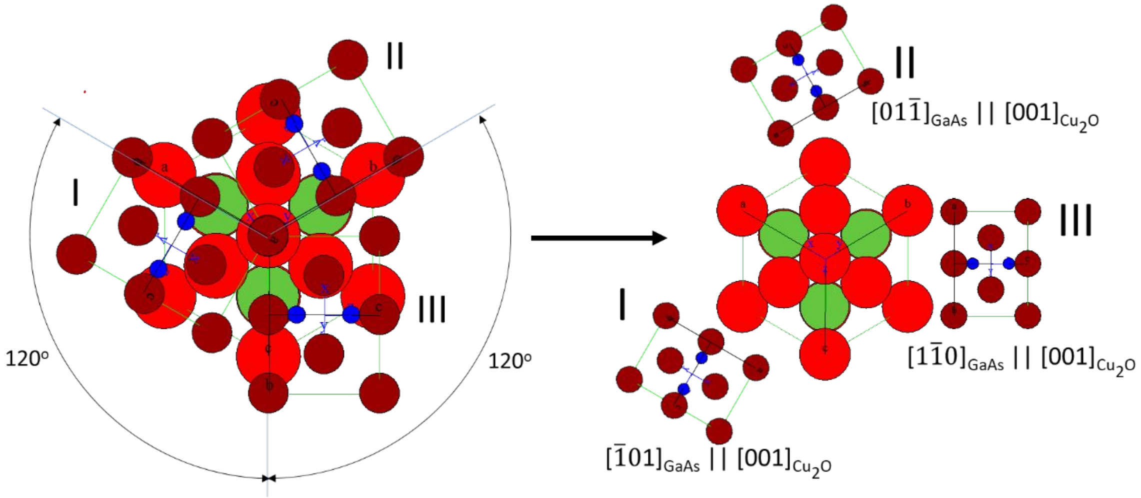

Figure S2. Schematic illustration of three possible orientations of Cu_2O grains grown onto GaAs (111)B system with $(111)_{GaAs}$ ǁ $(110)_{Cu2O}$ substrate / film relationship.

According to our Auger parameter (AP) calculations for the Cu 2p and Cu LMM peaks, AP = 916.6 + 932.2 = 1848.8 ± 0.25 eV, which is in good agreement with the literature for the Cu^{+} oxidation state [1] and the value of 1849.8 eV. [2] At the same time, we found that the copper state labeled on Cu2p core-level spectra as Cu^{2+} corresponds to diffused Cu^{2+} ions from solution into the GaAs substrate (BE ~ 932.8 eV). These ions have lower BE than can be expected for the pure oxide CuO_2 (BE ~ 933.6 eV). This supports the hypothesis that formation of the Cu_2O film starts from penetration of Cu atoms into the substrate, followed by redox process which subsequently facilitates film growth.

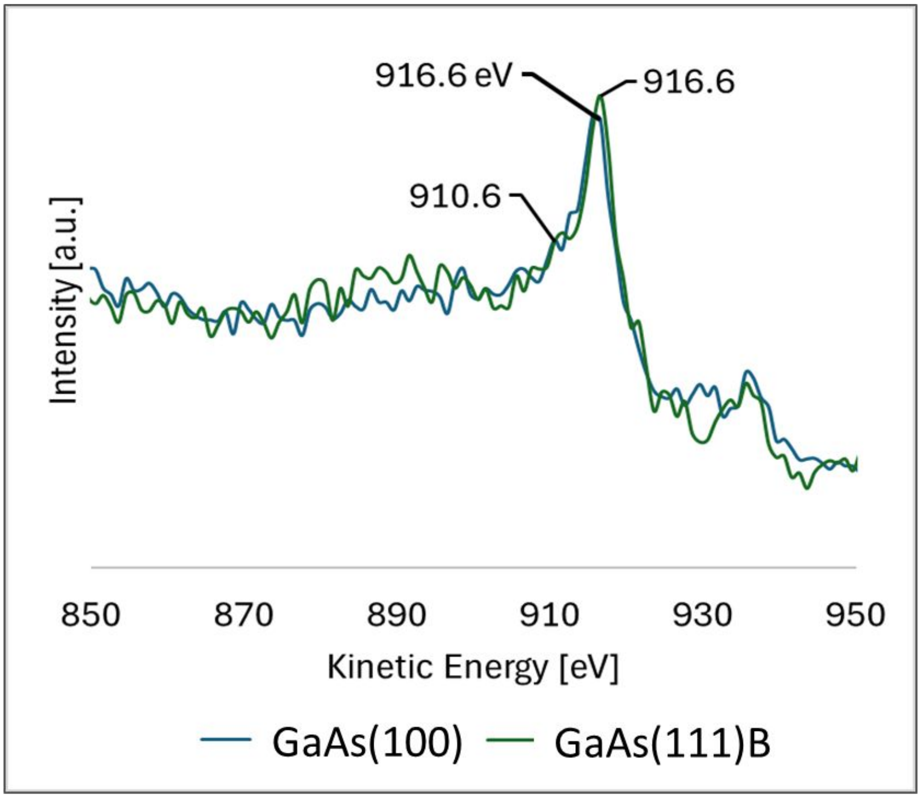

Figure S3. Cu LMM Auger line, obtained from the surface of Cu_2O films grown on GaAs (100) and GaAs (111) substrates.

1. X-ray Photoelectron Spectroscopy (XPS) Reference Pages: Copper. https://www.xpsfitting.com/2012/01/copper.html (accessed 2024-09-09).

2. Gahlot, S.; Dappozze, F.; Singh, D.; Ahuja, R.; Cardenas, L.; Burel, L.; Amans, D.; Guillard, C.; Mishra, S. Room-Temperature Conversion of $Cu_{2-x}Se$ to CuAgSe Nanoparticles to Enhance the Photocatalytic Performance of Their Composites with TiO_2. *Dalt. Trans.* **2020**, 49, 3580–3591.
